# Heat Shock Modulates Neutrophil Motility in Zebrafish

**DOI:** 10.1371/journal.pone.0084436

**Published:** 2013-12-19

**Authors:** Pui-ying Lam, Elizabeth A. Harvie, Anna Huttenlocher

**Affiliations:** 1 Program in Cellular and Molecular Biology, University of Wisconsin-Madison, Madison, Wisconsin, United States of America; 2 Department of Medical Microbiology and Immunology, University of Wisconsin-Madison, Madison, Wisconsin, United States of America; 3 Microbiology Doctoral Training Program, University of Wisconsin-Madison, Madison, Wisconsin, United States of America; 4 Department of Pediatrics, University of Wisconsin-Madison, Madison, Wisconsin, United States of America; Boston University, United States of America

## Abstract

Heat shock is a routine method used for inducible gene expression in animal models including zebrafish. Environmental temperature plays an important role in the immune system and infection progression of ectotherms. In this study, we analyzed the impact of short-term heat shock on neutrophil function using zebrafish (*Danio rerio*) as an animal model. Short-term heat shock decreased neutrophil recruitment to localized *Streptococcus iniae* infection and tail fin wounding. Heat shock also increased random neutrophil motility transiently and increased the number of circulating neutrophils. With the use of the translating ribosome affinity purification (TRAP) method for RNA isolation from specific cell types such as neutrophils, macrophages and epithelial cells, we found that heat shock induced the immediate expression of heat shock protein 70 (*hsp70*) and a prolonged expression of heat shock protein 27 (*hsp27*). Heat shock also induced cell stress as detected by the splicing of X-box binding protein 1 (*xbp1*) mRNA, a marker for endoplasmic reticulum (ER) stress. Exogenous expression of Hsp70, Hsp27 and spliced Xbp1 in neutrophils or epithelial cells did not reproduce the heat shock induced effects on neutrophil recruitment. The effect of heat shock on neutrophils is likely due to a combination of complex changes, including, but not limited to changes in gene expression. Our results indicate that routine heat shock can alter neutrophil function in zebrafish. The findings suggest that caution should be taken when employing a heat shock-dependent inducible system to study the innate immune response.

## Introduction

Fever is an evolutionarily conserved response during infection. In ectotherms such as fish, where regulation of body temperature depends on external sources, behavioral fever is often displayed as a result of infection, manifested as an acute change in thermal preference. Examples of this can be found in largemouth blackbass and bluegill sunfish which prefer elevated water temperatures after injection with killed *Aeromonas hydrophila* (*A.h*) [1]. Goldfish maintained at a febrile temperature had a higher survival rate after injection with live *A.h* [2]. Rainbow trout show an increased preferred temperature and enhanced expression of inflammatory cytokine interleukin-1β after bacterial lipopolysaccharide (LPS) injection [3]. Zebrafish also exhibit behavioral fever following viral infection, accompanied by upregulation of anti-viral genes [4]. Knowledge of what effect an elevated temperature has on immune cell function is limited. Several studies suggest that heat can affect neutrophil activation under some conditions. For example, heat exposure down-regulates TNFα signaling in suspended neutrophils but not in neutrophils interacting with fibronectin *in vitro* [5]. Heat inhibits LPS-induced neutrophil NF-κB activation in mice [6]. The physiological and molecular consequences of elevated body temperature on immune cell function *in vivo* are not clear. 

In addition to environmental temperature changes, short-term heat shock is a routine method for inducible gene expression with the use of heat shock protein promoters in various model organisms including mouse [7], zebrafish [8], *Drosophila* [9] and *Caenorhabditis elegans* [10,11]. Elevated temperature increases the expression of heat shock proteins (HSPs), which play important roles in innate and adaptive immunity (reviewed in 12–15). Heat stress can also induce the unfolded protein response (UPR), and immune systems rely on intact UPR functions (reviewed in 16,17). This study will address potential implications of the short-term heat shock procedure on neutrophil function.

In this paper, we focused on the effect of short-term temperature elevation on innate immune function using zebrafish (*Danio rerio*) as an animal model. The immune system of the zebrafish is highly conserved and has emerged as a powerful vertebrate disease model of both innate and adaptive immunity (reviewed in 18–20). Our data suggest that short-term heat shock affects innate immune function *in vivo* including a decrease in neutrophil recruitment to sites of infection and wound, and an increase in the mobilization of neutrophils into the circulation. This short-term heat shock, however, had no effect on larval survival with infection. Short-term heat shock increased neutrophil motility in a transient manner while the effect of heat shock on neutrophil wound response was long lasting. We showed that heat shock induced the expression of heat shock proteins such as Hsp70 and Hsp27 as well as the splicing of X-box binding protein 1 (*xbp1*) as part of the unfolded protein response. We adapted the translating ribosome affinity purification (TRAP) method for RNA isolation from specific cell types [21,22] in zebrafish and found that heat shock induced specific changes in gene expression in neutrophils, macrophages and epithelial cells. Neutrophil specific over-expression of Hsp70, Hsp27 and spliced Xbp1 did not recapitulate the observed heat shock phenotypes. These findings suggest against a cell autonomous role for these specific heat shock-induced genes on neutrophil function. Our findings suggest that caution should be taken when using the heat shock procedure to study innate immune responses since heat shock alone can induce changes in neutrophil function.

## Results and Discussion

### Heat shock induces changes in the innate immune response

**Figure 1:**
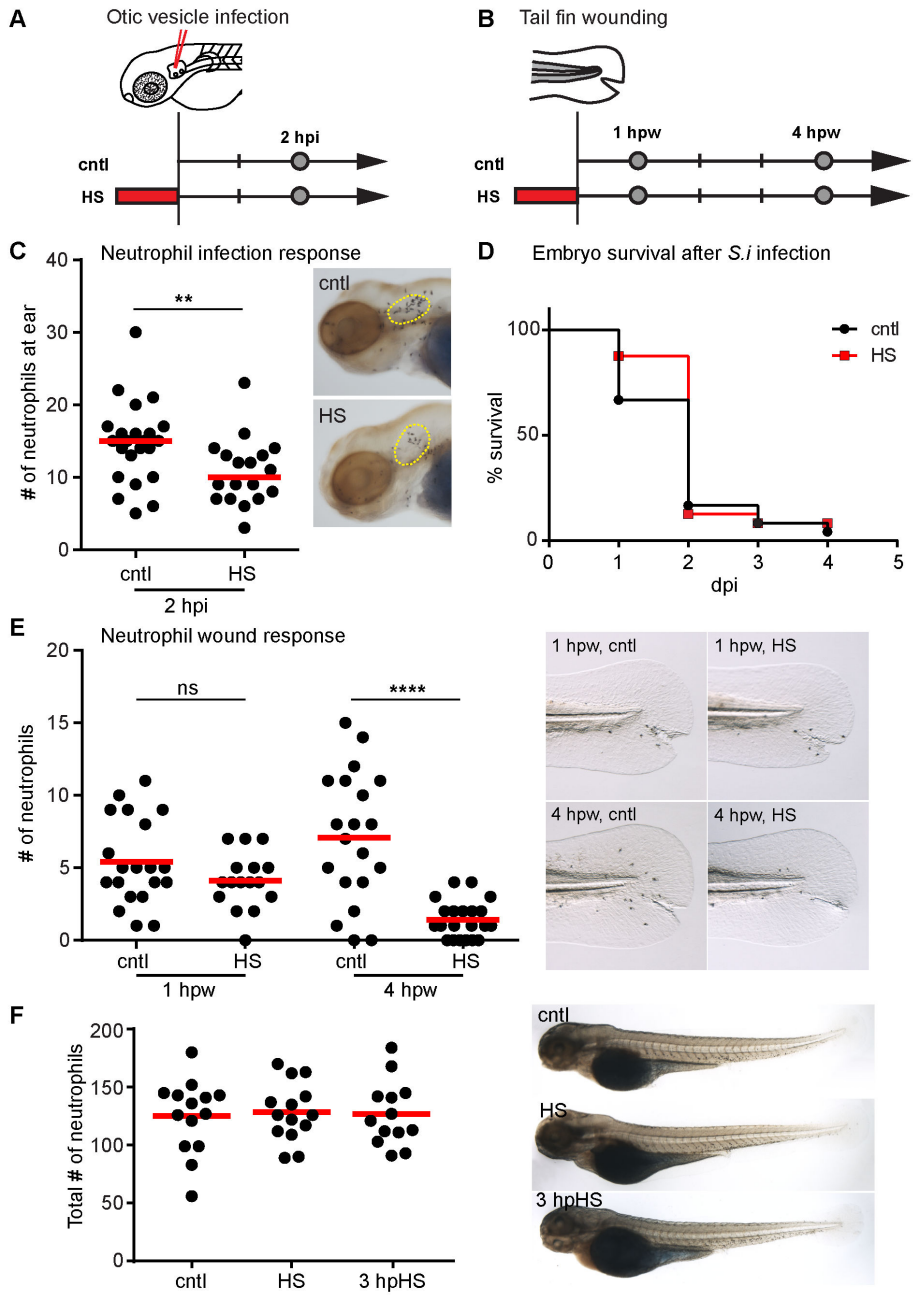
Heat shock effects on neutrophil recruitment. (A) Schematic illustration of the experimental setup for otic vesicle infection shown in C. (B) Schematic illustration of the experimental setup for tail fin wounding shown in E. The red rectangle indicates heat shock at 38-39°C for 1 hour. Grey circles indicate the time point for neutrophil counting. (C) Quantification of neutrophils at the otic vesicle (yellow dotted line) in control (cntl) and heat shocked (HS) larvae at 2 hours post infection (2 hpi). Larvae were injected with 100 CFU of Streptococcus iniae (S.i) into the otic vesicle (ear) at 3 days post fertilization (dpf). HS larvae showed decreased neutrophil recruitment compared with controls. **P<0.01 (two-tailed, unpaired t-test). (Right panel) Representative images of Sudan Black-stained larvae. Lateral view of the head of larvae at 2 hpi. (D) Survival of control (cntl) and heat shocked (HS) larvae infected with S.i. in the ear over time. Control and HS larvae showed a similar survival rate. dpi; days post infection. (E) Quantification of neutrophils at wounds in control (cntl) and heat shocked (HS) larvae at 1 and 4 hour post wounding (hpw). HS larvae showed fewer neutrophils at wounds at 4 hpw compared with controls. ****P<0.0001; ns, not significant (two-tailed, unpaired t-test). (Right panel) Representative images of Sudan Black-stained larvae. Lateral view of the tail fin of larvae at 3 dpf. (F) Quantification of neutrophils in whole larvae for control (cntl), heat shocked (HS) and 3 hours post heat shocked (3 hpHS) larvae. (Right panel) Representative images of Sudan Black-stained larvae. Data are representative of at least three experiments.

Zebrafish are normally maintained at 28.5°C. Heat shock (HS) was performed at 38-39°C for 1 hour in a water bath. To assess the effect of heat shock on immune cells, we performed the previously established methods of *Streptococcus iniae* (*S.i*) otic vesicle infections (Figure 1A) [23] and tail fin wound assays (Figure 1B) [24] on zebrafish larvae at 3 days post fertilization (dpf). At this stage of development, adaptive immunity has not sufficiently matured [25,26], allowing for the direct study of innate immunity in isolation. Neutrophils, as the first responders to sites of infection or wounds (reviewed in 27), are an important part of the innate immune response. Larvae were infected with *S.i* or injected with a PBS control in the otic vesicle and fixed at 2 hours post infection (hpi) for Sudan Black staining and quantification of neutrophil recruitment. There was a decrease in neutrophil recruitment to the site of *S.i.* otic infection in HS larvae compared to controls (Figure 1C). Since it has been shown that tilapia are more susceptible to *S.i* infection at higher temperatures [28], we then tested if short-term HS can affect the survival of zebrafish larvae after *S.i* infection. In our system, short-term HS for 1 hour did not alter survival 4 days post infection (Figure 1D). HS also decreased neutrophil recruitment to the tail fin wound at 4 hours post wounding (hpw) when compared to controls (Figure 1E). Since HS did not significantly change the total number of neutrophils (Figure 1F), the effect was likely due to a change in neutrophil behavior after HS. Taken together, our results suggest that HS impairs neutrophil recruitment to sites of infection and tissue damage.

### Heat shock affects neutrophil motility

**Figure 2:**
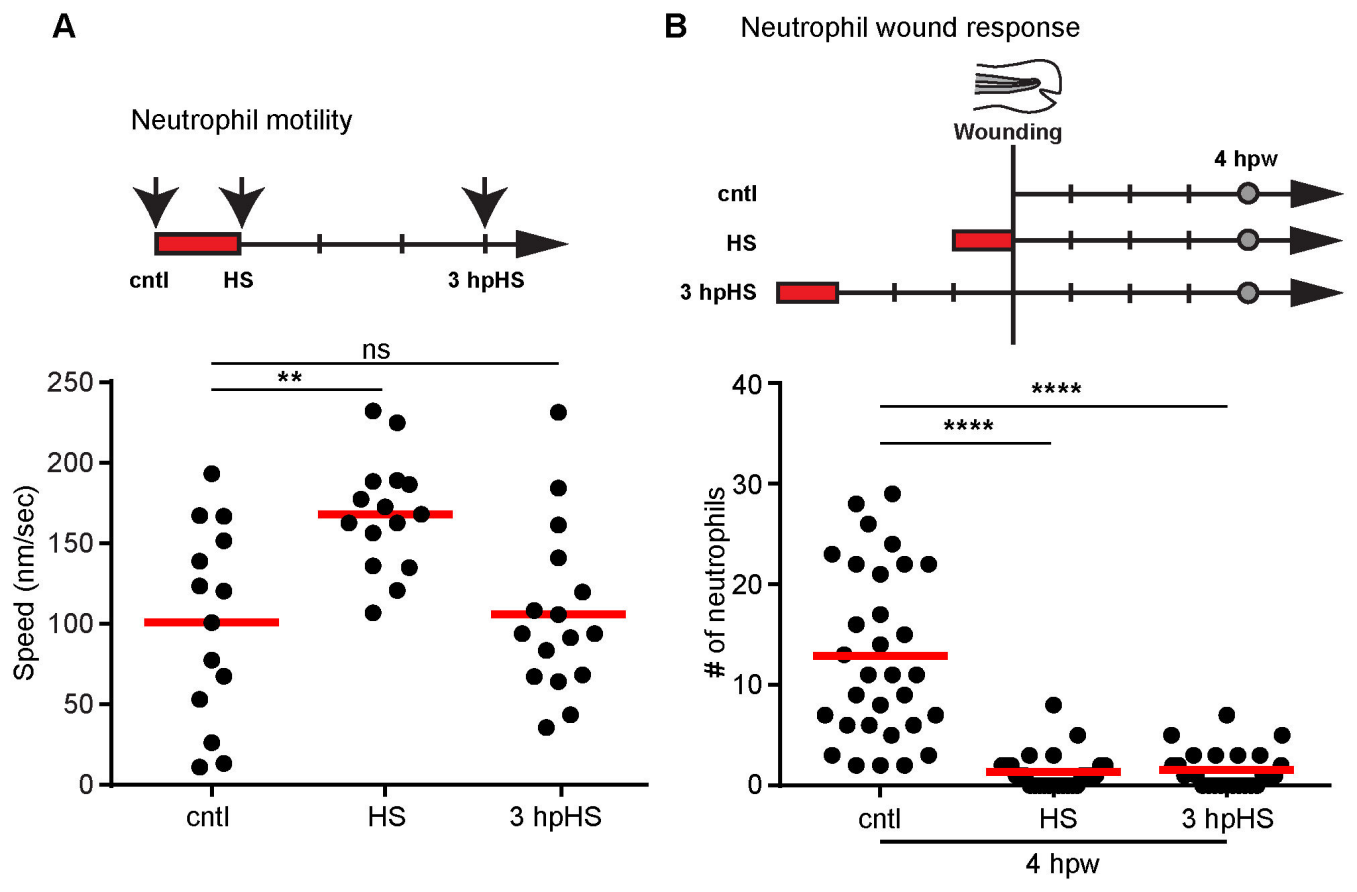
Heat shock induces transient changes in neutrophil random motility and sustained effects on recruitment to wounds. (A) (Top) Schematic illustration of the experimental setup for quantification of neutrophil speed. The red rectangles indicate heat shock at 38-39°C for 1 hour. Arrows indicate the time point for neutrophil live imaging in the same larva before heat shock (cntl), right after heat shock (HS) and at 3 hours post heat shock (3 hpHS). (Bottom) Scatter plot showing the mean speed of Tg(mpx:dendra2) neutrophils at time points indicated. Neutrophils were tracked in 3 dimensions (3D) using Image J software and the MTrackJ plugin. HS larvae showed increased neutrophil speed compared with controls. At 3 hpHS, the speed of neutrophils went back to control levels. **P<0.01; ns, not significant (one way ANOVA with Dunn’s multiple comparison test). (B) (Top) Schematic illustration of the experimental setup for tail fin wounding. Larvae were wounded without heat shock (cntl), right after heat shock (HS) or at 3 hours post heat shock (3 hpHS). Larvae were then fixed at 4 hours post wounding (hpw) for quantification of neutrophils (grey circles). (Bottom) Quantification of neutrophils at tail fin wounds at 4 hpw. The effect of HS on neutrophil wound response persisted even when the wound was induced at 3 hpHS. ****P<0.0001 (one way ANOVA with Dunn’s multiple comparison test). Data are representative of at least three experiments.

It has previously been reported that isolated human neutrophils display an increase in motility at higher temperatures [29]. To further characterize the effects of heat shock, we analyzed the speed of neutrophils undergoing random motility in the head region of zebrafish larvae. We observed an increase in neutrophil speed immediately after HS followed by a return to baseline motility at 3 hours post heat shock (hpHS) (Figure 2A). By contrast, the effect of HS on neutrophil wound recruitment was long lasting. When larvae were wounded at 3 hpHS and then assayed four hours later, there was a significant decrease in the number of neutrophils at the tail wound site at 4 hpw (Figure 2B). 

### Heat shock increases neutrophil mobilization

**Figure 3:**
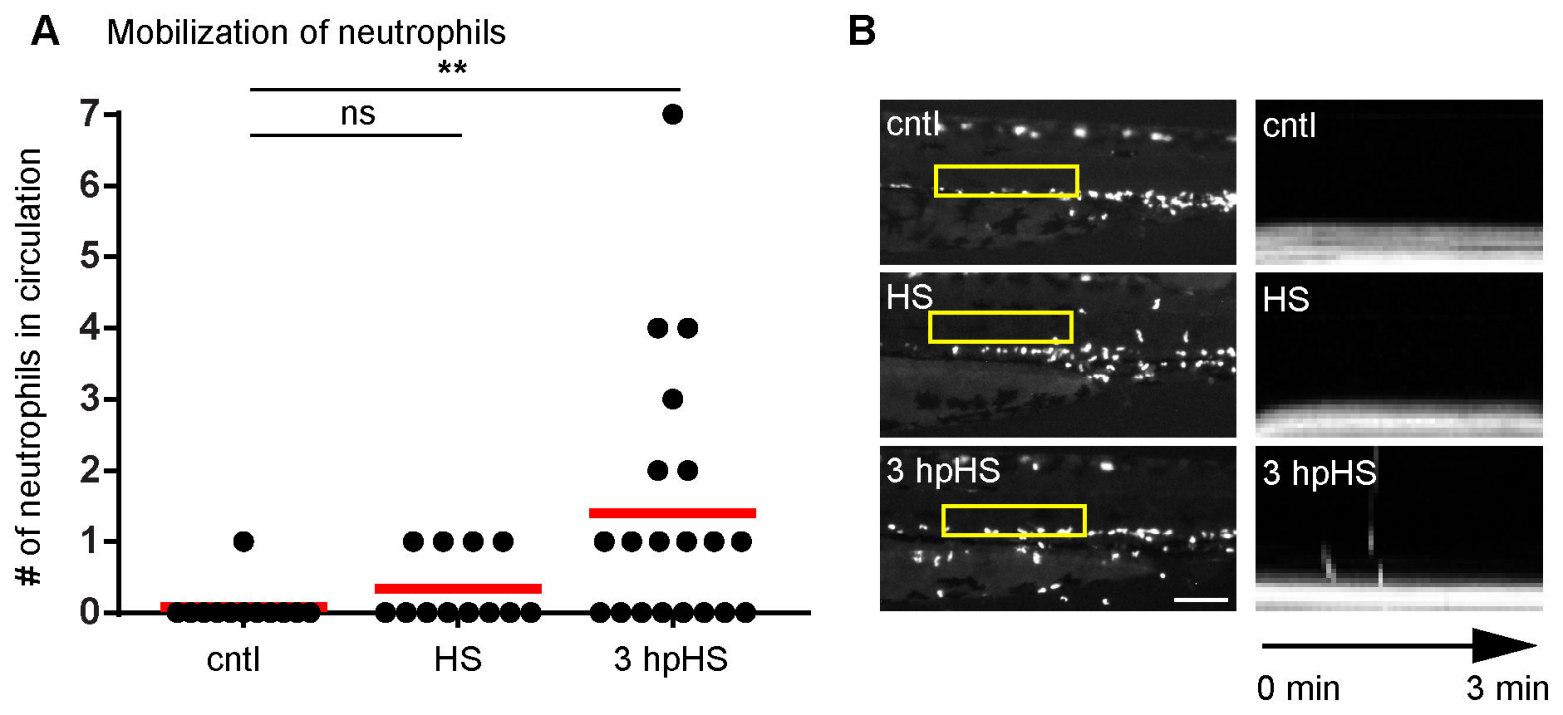
Heat shock induces neutrophil mobilization. (A) Quantification of circulating neutrophils in larvae at 3 dpf. There was an increase in circulating neutrophils at 3 hpHS. **P<0.01; ns, not significant (one way ANOVA with Dunn’s multiple comparison test). (B) (Left panel) Yellow box indicates area where kymograph was generated. Fluorescent signal in boxed region was stacked vertically into a one-dimensional line at each time point. Scale bar = 100 µm. (Right panel) Kymograph of 3 minute movies with a 3 second interval indicating the presence or absence of circulating neutrophils is shown. Data are representative of at least three experiments.

Since HS impaired neutrophil wound recruitment without affecting global numbers of neutrophils (Figure 1F), we next tested if we could detect a change in neutrophil distribution induced by HS. Previous work has established that neutrophils in zebrafish larvae reside outside the vasculature, in a region known as the caudal hematopoietic tissue (CHT) and only mobilize into the circulation upon infection [30], wounding [31] or with leukocyte adhesion deficiency [32]. Using the zebrafish transgenic lines (*Tg*(*mpx:dendra2*) or *Tg*(*mpx:mCherry*)), expressing fluorescent proteins specifically in neutrophils, we were able to perform live imaging of circulating neutrophils *in vivo*. We observed an increase in circulating neutrophils, particularly at 3 hpHS (Figure 3A and 3B). This may indicate that HS leads to either activation of the neutrophils or impaired retention of neutrophils within the CHT.

### Heat shock induces changes in gene expression

We were interested in exploring the idea that heat shock-induced changes in gene expression might be responsible for the modification in neutrophil behavior. We looked at changes in gene expression immediately after HS and at 3 hpHS to assess the immediate-early and longer-term changes in gene expression. We focused on three candidate genes, *hsp70*, *hsp27* and the splicing of *xbp1*, due to their roles in immune function. Cell stress such as heat shock, infection, fever, inflammation, malignancy or autoimmunity can induce heat shock protein (HSP) synthesis (reviewed in 33). HSP70 is involved in both innate and adaptive immunity and has been extensively reviewed [15,34,35,36,37]). The role of HSP70 in inflammation is controversial. Recent studies suggest that it has pro-inflammatory functions [38] as well as anti-inflammatory functions [39]. HSP27 has been shown to function in different cellular processes such as protein folding, actin remodeling and in the reduction of oxidative stress (reviewed in 40). HSP27 binds to the barbed end of actin filaments and inhibits actin polymerization [41–43]. HSP27 has also been shown to be important for cell motility in human cancer cells [44]. It has previously been reported that HSP27 regulates neutrophil chemotaxis, however, this role is independent of its role in regulating actin reorganization [45]. Although research on zebrafish Hsp27 is limited, it has been shown to have similar functions and to be regulated like mammalian HSP27 [46,47]. 

Upon ER stress induced unfolded protein response, the mRNA encoding the X-box-binding protein-1 (XBP1) is spliced by IRE1 to generate a more potent transcription factor XBP1S [48]. XBP1 protects *Caenorhabditis elegans* during the activation of its innate immune response upon infection with pathogenic bacteria [49]. It has been shown that toll-like receptor activation leads to the production of spliced XBP1 in mouse macrophages, which is required for optimal production of proinflammatory cytokines [50]. XBP1 has also been linked to intestinal inflammation [51]. The splicing of *xbp1* in response to ER stress is conserved in zebrafish [52].

**Figure 4:**
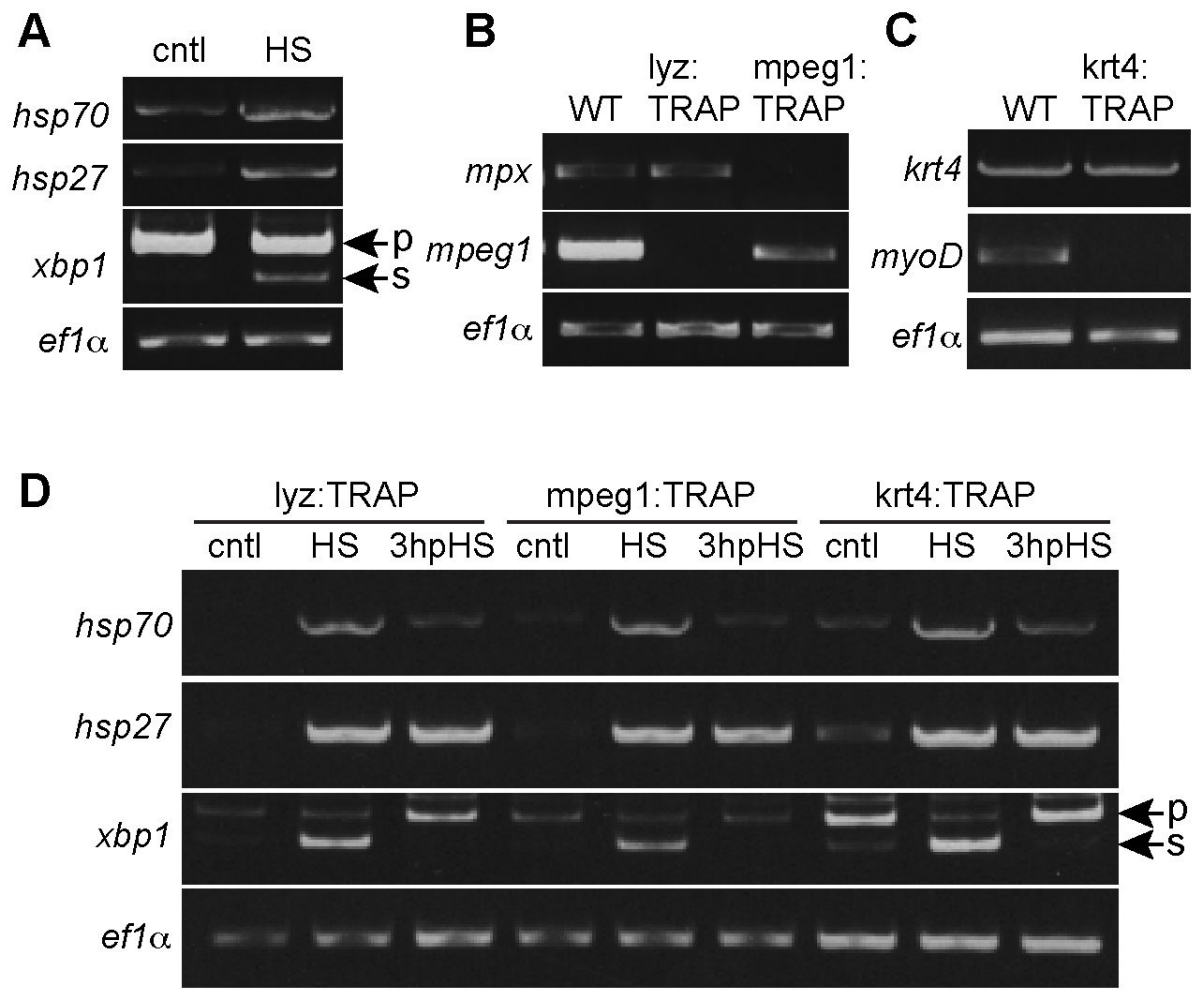
Heat shock induces changes in gene expression. (A) RT-PCR analysis of *hsp70*, *hsp27* and splicing of *xbp1* (p, pre-sliced; s, spliced) in control (cntl) or heat shock (HS) larvae. (B-C) RT-PCR analysis of the enrichment of RNA from neutrophils (lyz:TRAP), macrophages (mpeg1:TRAP) or epithelial cells (krt4:TRAP) from *Tg*(*lyz:EGFP-L10a*), *Tg*(*mpeg1:EGFP-L10a*) or *Tg*(*krt4:EGFP-L10a*) larvae, respectively. Whole larvae RNA (WT) or translating ribosome affinity purification (TRAP) RNA was used and specific markers for neutrophils (*mpx*), macrophages (*mpeg1*), epithelial cells (*krt4*) and muscle cells (*myoD*) were tested. (D) Heat shock (HS) induced expression of *hsp70*, *hsp27* and splicing of *xbp1* (p, pre-sliced; s, spliced) in neutrophils (lyz:TRAP), macrophages (mpeg1:TRAP) and epithelial cells (krt4:TRAP). Expression of *hsp27* persisted at 3 hours post heat shock (3 hpHS) while expression of *hsp70* and splicing of *xbp1* returned to control levels at 3 hpHS. Data are representative of at least two experiments.

We found that heat shock induced a global increase in *hsp70* and *hsp27* expression as well as the splicing of x*bp1* (Figure 4A). These specific heat shock proteins are normally expressed during development in certain tissue but not others. *hsp70*, for example, is normally expressed during lens development in zebrafish [53], while *hsp27* is normally expressed in skeletal and cardiac muscle tissues [54]. To assess changes in gene expression in specific cell types and to identify translationally active mRNAs from steady-state total mRNAs [55], we adapted the translating ribosome affinity purification (TRAP) method that was previously developed for murine models [21,22]. This method involves the expression of EGFP-tagged mouse ribosomal protein L10a in defined cell populations, allowing characterization of actively translating genes in specific cell types. Since zebrafish L10a shares considerable homology with its mouse counterpart, we predicted the same construct could be employed for zebrafish TRAP. We generated three transgenic fish lines where EGFP-L10a is specifically expressed in neutrophils (lyz:TRAP), macrophages (mpeg1:TRAP) or epithelial cells (krt4:TRAP). The enriched mRNAs isolated from these transgenic lines were then analyzed. As expected, the lyz:TRAP and mpeg1:TRAP lines showed the expression of either the neutrophil marker (*mpx*) or macrophage marker (*mpeg1*), respectively (Figure 4B). In the krt4:TRAP line, we detected the expression of the epithelial marker *krt4* but not the muscle marker *myoD* (Figure 4C). We then used these transgenic lines to assess whether or not HS induced the expression of *hsp70*, *hsp27* and the splicing of *xbp1* specifically in these subsets of cells. Using the TRAP method, we observed that HS did indeed induce the expression of *hsp70* and *hsp27* in neutrophils, macrophages and epithelial cells (Figure 4D). At 3 hpHS, expression of *hsp27* persisted while the expression of *hsp70* returned to baseline levels. Heat shock induced splicing of *xbp1* occurred in all three cell populations tested but appears to be short lived since there was no *xbp1* splicing detectable at 3 hpHS (Figure 4D).

### The role of Hsp70, Hsp27 and spliced Xbp1 in neutrophil motility

**Figure 5:**
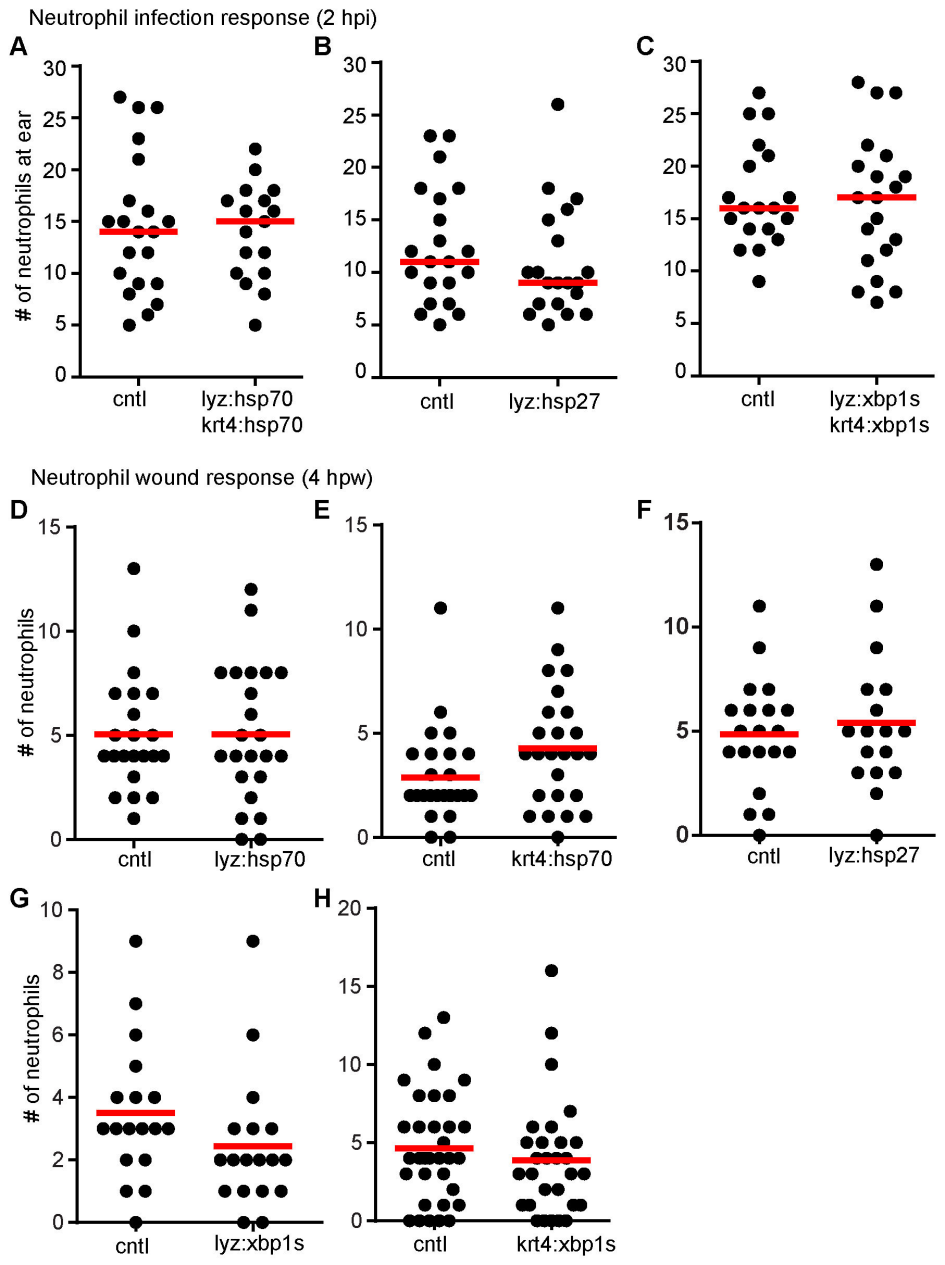
Expression of Hsp27, Hsp70 or spliced Xbp1 in neutrophils or epithelial cells does not alter neutrophil recruitment. (A-C) Quantification of neutrophils at the otic vesicle (ear) of larvae at 2 hours post infection (hpi) with 100 CFU *S.i.* ear infection. Experiments were performed on the larvae of transgenic lines *Tg*(*lyz:hsp70-2A-EGFP*) x *Tg*(*krt4:hsp70-2A-mCherry*) (A), *Tg*(*lyz:hsp27-2A-mCherry*) (B) or *Tg*(*lyz:xbp1s-2A-EGFP*) x *Tg*(*krt4:xbp1s-2A-mCherry*) (C) at 3 dpf. Controls (cntl) were siblings with no transgene expression. Expression of Hsp27, Hsp70 and spliced Xbp1 (Xbp1s) did not have a significant effect on neutrophil recruitment to ear infection. (D-H) Quantification of neutrophils at tail fin wounds at 4 hpw. Experiments were performed on *Tg*(*lyz:hsp70-2A-EGFP*) (D), *Tg*(*krt4:hsp70-2A-mCherry*) (E), *Tg*(*lyz:hsp27-2A-mCherry*) (F), *Tg*(*lyz:xbp1s-2A-EGFP*) (G) or *Tg*(*krt4:xbp1s-2A-mCherry*) (H) larvae at 3 dpf. Controls (cntl) were siblings with no transgene expression. Expression of Hsp27, Hsp70 and spliced Xbp1 (Xbp1s) did not have a significant effect on neutrophil numbers at the wound at 4 hpw. Data are representative of at least two experiments.

In an attempt to determine if elevated levels of Hsp70, Hsp27 and spliced Xbp1 directly affect innate immune functions, we specifically expressed these proteins in neutrophils and/or epithelial cells. Expression of Hsp70 or spliced Xbp1 in neutrophils and epithelial cells did not affect the neutrophil response to *S.i.* otic infection (Figure 5A and 5C). Similar results were observed when Hsp27 was specifically expressed in neutrophils (Figure 5B). We next asked if over-expression of Hsp70, Hsp27 or spliced Xbp1 individually in neutrophils or epithelial cells could reproduce the HS phenotypes. As seen in Figure 5D-5H, this approach did not reproduce the HS phenotype of altering neutrophil recruitment. However, we cannot rule out the involvement of these genes and must consider that constitutive expression of HSPs and spliced Xbp1 may not faithfully recapitulate the short-term HS condition. Furthermore, the HS effects on neutrophil function may not be neutrophil cell autonomous. For example, it has been shown that elevated body temperatures can enhance LPS-induced proinflammatory cytokine TNFα production in macrophages [56]. Similarly, the effect of heat shock on neutrophil function is likely due to a combination of differential gene expression within neutrophils as well as in other cell types that act to guide or influence the behavior of neutrophils. 

Our results show that the effect of heat shock on the innate immune response should be taken into consideration when using heat shock as a tool for inducible gene expression.

This is particularly important in the zebrafish field since heat shock is routinely used for temporal control of gene expression [8,57–59], and the Hsp70 promoter is commonly used in the zebrafish community. As the expression of *hsp27* persisted long after the initial heat shock, researchers may want to explore the use of the Hsp27 promoter for inducible gene expression in zebrafish, keeping in mind its endogenous tissue expression [60]. 

### Conclusion

Our data suggest that short-term HS results in changes in neutrophil behavior including increased motility and mobilization but decreased response to infection and wounding. HS induces changes in gene expression including *hsp70* and *hsp27* and in the splicing of *xbp1*. These findings suggest that caution should be taken when employing a HS-dependent inducible system to study the innate immune system. The effect of long-term temperature increase on innate immunity will require further investigation.

## Materials and Methods

### Ethics Statement

All animal studies were approved by the University of Wisconsin – Madison Animal Care and Use Committee and performed in accordance with the guidelines.

### Heat shock, tail fin wounding, otic vesicle injection and Sudan Black staining

Heat shock was performed in a water bath at 38-39°C for 1 hour. For tail fin wounding, larvae at 3 dpf were anesthetized using 0.2 mg/mL tricaine and wounded with a 33 gauge needle. Preparation of *Streptococcus iniae* and microinjection of bacteria into zebrafish larvae has been previously described [23]. 1 nl volume of 100 CFU of *S. iniae* wild-type strain 9117 (or a PBS control) was microinjected into the otic vesicle of larvae at 3 dpf. Larvae were then fixed at the time points indicated with 4% formaldehyde overnight at room temperature and stained with Sudan Black as described previously [61].

### Purification of mRNA from TRAP zebrafish larvae and RT-PCR

A protocol previously used for TRAP mRNA purification from mouse tissue [21] was adapted for zebrafish larvae with slight modifications. Briefly, a glass dounce homogenizer was used to homogenize the larvae. Purified rabbit anti-GFP antibody (Invitrogen A11122) was used for immunoprecipitation. After immunoprecipitation and high-salt polysome buffer washing steps, RNA was purified using an RNeasy Mini Kit (Qiagen) with in-column DNase digestion. RT-PCR was performed using the OneStep RT-PCR Kit (Qiagen) according to the manufacturer’s instruction. Primers used to amplify *ef1α*, *mpx* [62] and *xbp1* [52] have been described previously. The other primer sequences used in this study were as follow:

**hsp27-F** 5’-CGGATCCATGGCCGAGAGACGCATC-3’
**hsp27-R** 5’-TTATTTTGTGGTGCTGACGG-3’
**hsp70-F** 5’-CACCCAGCTATGTTGCCTTCAC-3’
**hsp70-R** 5’-CACCATGCGGTTGTCAAAGTCC-3’
***krt4*-F** 5’-CTATGGAAGTGGTCTTGGTGGAGG-3’
***krt4*-R** 5’-CCTGAAGAGCATCAACCTTGGC-3’
***mpeg1*-F** 5’-CTTTAATTCAGAGCCACGGAGGAGC-3’
***mpeg1* R** 5’-GTAGACAACCCTAAGAAACCACAGG-3’
***myoD*-F** 5’-CCTTGCTTCAACACCAACGACATG-3’
***myoD*-R** 5’-GTCATAGCTGTTCCGTCTTCTCGTC-3’

PCR products were analyzed using 1% agarose electrophoresis with the exception of *xbp1* RT-PCR in which 8% polyacrylmide gel made in TAE buffer was used.

### DNA injection

All DNA expression vectors contained the zebrafish lysozyme C (*lyz*) promoter for neutrophil expression [24,63], *mpeg1* promoter for macrophage expression [23], or *krt4* promoter for epithelial cell expression [64,65]. To facilitate the production of multiple protein products from a single transgene, a viral 2A peptide linker sequence was used [66]. All expression vectors contain minimal Tol2 elements for efficient integration [67] and an SV40 polyadenylation sequence (Clontech Laboratories, Inc). The following constructs were generated: *lyz-EGFP-L10a, mpeg1-EGFP-L10a*, *krt4-EGFP-L10a, lyz-hsp27-2A-mCherry* (Zebrafish *hsp27* (ATCC 10809422, accession BC097148)), *lyz-hsp70-2A-EGFP* (Zebrafish *hsp70* (Open Biosystems Clone Id:6789418, accession BC056709)), *krt4-hsp70-2A-mCherry*, *lyz-xbp1-2A-EGFP* (Zebrafish spliced *xbp1* (Open Biosystems Clone Id:3816043, accession BC044134)) and *krt4-xbp1-2A-mCherry*. Expression of constructs was obtained by injecting 3 nL of solution containing 12.5 ng/μL of DNA plasmid and 17.5 ng/μL *in vitro* transcribed (Ambion) Tol2 transposase mRNA into the cytoplasm of one-cell stage embryo. 

### Live imaging and image quantification

Larvae at 3 dpf were anesthetized using 0.2 mg/mL tricaine and mounted on a glass-bottom dish with 1% low melt agarose for live imaging. Time-lapse fluorescence images were acquired using a confocal microscope (FluoView FV1000, Olympus) using a NA 0.75/20x objective. Z-stack images were collected for 30 minutes with 1 minute intervals. 3-dimentional tracking of neutrophils has been previously described [24]. Quantification of circulating neutrophils has been previously described [30]. Briefly, *Tg*(*mpx:mCherry*) or *Tg*(*mpx:dendra2*) were used and neutrophils that circulate through the posterior cardinal vein were scored in individual 3 minute movies with 3 second intervals using a Nikon SMZ-1500 zoom microscope equipped with epifluorescence and a CoolSnap ES camera (Roper Scientific, Duluth, GA).

### Statistics

Experimental results were analyzed with Prism version 4 (GraphPad Software) statistical software. The resulting *P* values are included in the figure legends for each experiment.
